# Supplementary material for: Genomic Diversity in Sporadic Breast Cancer in a Latin American Population
Source: Genes (Basel). 2020 Oct 28;11(11):1272. doi: 10.3390/genes11111272 (PMC7716199; doi:10.3390/genes11111272)
Supplement: Supplementary file 1 [file genes-11-01272-s001.zip › Supplementary Table S2.docx]

Supplementary Table S2. Worldwide haplotype distribution of ESR1, VDR, BRCA2 and BRCA1 genes

|  | | Asian | | African | | European | | | Latin American | | | | Uruguay | |
| --- | --- | --- | --- | --- | --- | --- | --- | --- | --- | --- | --- | --- | --- | --- |
| CHR / HAP | | CHB | CHS | GWD | YRI | IBS | TSI | CEU | CLM | MXL | PEL | PUR | P | C |
| 6 | rs1514347\|rs6912184 | | | | | | | | | | | | | |
| ESR1 | CA | 0.490 | 0.471 | 0.849 | 0.829 | 0.789 | 0.757 | 0.797 | 0.712 | 0.671 | 0.575 | 0.783 | 0.779 | 0.789 |
|  | TG | 0.490 | 0.490 | 0.079 | 0.053 | 0.173 | 0.210 | 0.154 | 0.233 | 0.273 | 0.373 | 0.163 | 0.169 | 0.148 |
|  | TA | ------ | ------ | ------ | 0.052 | 0.028 | 0.019 | 0.036 | 0.038 | 0.040 | 0.032 | 0.034 | 0.029 | 0.014 |
|  | CG | 0.015 | 0.034 | 0.063 | 0.066 | ------ | 0.014 | 0.012 | 0.170 | 0.016 | 0.020 | 0.020 | 0.022 | 0.049 |
| 12 | rs7975232\|rs1544410\|rs2239182\|rs2239179 | | | | | | | | | | | | | |
| VDR | ATCC | 0.025 | 0.031 | 0.168 | 0.219 | 0.348 | 0.334 | 0.437 | 0.177 | 0.133 | 0.092 | 0.344 | 0.276 | 0.265 |
|  | CCTT | 0.525 | 0.543 | 0.172 | 0.117 | 0.295 | 0.355 | 0.310 | 0.388 | 0.431 | 0.731 | 0.316 | 0.338 | 0.362 |
|  | ACTT | 0.230 | 0.169 | 0.244 | 0.158 | 0.061 | 0.089 | 0.071 | 0.085 | 0.185 | 0.044 | 0.088 | 0.107 | 0.124 |
|  | CCCT | ------ | ------ | 0.148 | 0.224 | 0.067 | 0.037 | 0.045 | 0.053 | 0.026 | ------ | 0.044 | 0.040 | 0.032 |
|  | CCCC | 0.175 | 0.207 | 0.025 | 0.046 | 0.067 | 0.019 | 0.044 | 0.041 | 0.137 | 0.033 | 0.048 | 0.036 | 0.045 |
|  | ATTT | 0.021 | 0.016 | 0.045 | 0.046 | 0.078 | 0.061 | 0.036 | 0.062 | 0.041 | 0.015 | 0.042 | 0.049 | 0.047 |
|  | ACCC | ------ | ------ | 0.050 | 0.017 | 0.041 | 0.058 | 0.030 | 0.115 | 0.026 | 0.032 | 0.040 | 0.107 | 0.072 |
|  | ATCT | ------ | ------ | ------ | 0.030 | ------ | 0.021 | 0.015 | ------ | ------ | 0.020 | 0.023 | 0.018 | 0.013 |
|  | ACCT | ------ | ------ | 0.141 | 0.134 | 0.029 | 0.026 | ------ | 0.055 | 0.021 | ------ | 0.045 | 0.018 | 0.029 |
| 13 | rs206081\|rs11571787\|rs4942505 | | | | | | | | | | | | | |
| BRCA2 | CTT | 0.189 | 0.181 | 0.389 | 0.319 | 0.266 | 0.308 | 0.321 | 0.367 | 0.359 | 0.361 | 0.293 | 0.295 | 0.309 |
|  | CTC | 0.250 | 0.267 | 0.248 | 0.271 | 0.262 | 0.262 | 0.304 | 0.340 | 0.320 | 0.253 | 0.317 | 0.305 | 0.292 |
|  | TTC | 0.224 | 0.190 | 0.124 | 0.243 | 0.257 | 0.196 | 0.226 | 0.186 | 0.148 | 0.165 | 0.183 | 0.196 | 0.173 |
|  | CGT | 0.337 | 0.362 | 0.239 | 0.167 | 0.215 | 0.234 | 0.149 | 0.106 | 0.172 | 0.222 | 0.207 | 0.205 | 0.226 |
| 17 | rs8176193\|rs16942\|rs8176092 | | | | | | | | | | | |  |  |
| BRCA1 | CTT | 0.638 | 0.624 | 0.748 | 0.805 | 0.626 | 0.621 | 0.637 | 0.559 | 0.617 | 0.627 | 0.692 | 0.617 | 0.636 |
|  | TCG | 0.362 | 0.376 | 0.239 | 0.186 | 0.350 | 0.379 | 0.363 | 0.441 | 0.383 | 0.367 | 0.308 | 0.369 | 0.356 |
|  | CCT | ------ | ------ | ------ | ------ | ------ | ------ | ------ | ------ | ------ | ------ | ------ | 0.014 | 0.007 |
